# Translation of artemether–lumefantrine treatment policy into paediatric clinical practice: an early experience from Kenya

**DOI:** 10.1111/j.1365-3156.2007.01980.x

**Published:** 2008-01

**Authors:** D Zurovac, J Njogu, W Akhwale, D H Hamer, R W Snow

**Affiliations:** 1 Malaria Public Health and Epidemiology Group, KEMRI/Wellcome Trust Research ProgrammeNairobi, Kenya; 2 Centre for Tropical Medicine, University of Oxford, John Radcliffe HospitalOxford, UK; 3 Division of Malaria Control, Ministry of HealthNairobi, Kenya; 4 Center for International Health and Development, Boston University School of Public HealthBoston, MA, USA; 5 Section of Infectious Diseases, Department of Medicine, Boston University School of MedicineBoston, MA, USA

**Keywords:** malaria, change of treatment, sulfadoxine–pyremethamine, artemether–lumefantrine, health facility evaluation, Kenya

## Abstract

**Objective:**

To describe the quality of outpatient paediatric malaria case-management approximately 4–6 months after artemether–lumefantrine (AL) replaced sulfadoxine–pyrimethamine (SP) as the nationally recommended first-line therapy in Kenya.

**Methods:**

Cross-sectional survey at all government facilities in four Kenyan districts. Main outcome measures were health facility and health worker readiness to implement AL policy; quality of antimalarial prescribing, counselling and drug dispensing in comparison with national guidelines; and factors influencing AL prescribing for treatment of uncomplicated malaria in under-fives.

**Results:**

We evaluated 193 facilities, 227 health workers and 1533 sick-child consultations. Health facility and health worker readiness was variable: 89% of facilities stocked AL, 55% of health workers had access to guidelines, 46% received in-service training on AL and only 1% of facilities had AL wall charts. Of 940 children who needed AL treatment, AL was prescribed for 26%, amodiaquine for 39%, SP for 4%, various other antimalarials for 8% and 23% of children left the facility without any antimalarial prescribed. When AL was prescribed, 92% of children were prescribed correct weight-specific dose. AL dispensing and counselling tasks were variably performed. Higher health worker's cadre, in-service training including AL use, positive malaria test, main complaint of fever and high temperature were associated with better prescribing.

**Conclusions:**

Changes in clinical practices at the point of care might take longer than anticipated. Delivery of successful interventions and their scaling up to increase coverage are important during this process; however, this should be accompanied by rigorous research evaluations, corrective actions on existing interventions and testing cost-effectiveness of novel interventions capable of improving and maintaining health worker performance and health systems to deliver artemisinin-based combination therapy in Africa.

## Introduction

Artemisinin-based combination therapies (ACTs) are a major breakthrough in the clinical management of malaria. By 2006, 39 countries in Africa had revised their national drug policies to incorporate ACTs into malaria case-management guidelines (Olumese 2006). However, the implementation of effective case-management using ACTs faces a number of challenges, notably complex ordering and financing procedures, unfamiliarity with the drugs among prescribers and their introduction into weak health systems with suboptimal drug management and clinical practices (Rowe *et al.* 2000, 2001, 2003; Font *et al.* 2001; Nshakira *et al.* 2002; Armstrong Schellenberg *et al.* 2004; Eriksen *et al.* 2007). Therefore, despite their promise, new ACT drugs may not reach patients who need them and if they do, the challenge remains to ensure adequate clinical practices. There are very few studies that have examined the operational use of ACTs following national policy change (Zurovac *et al.* 2005a, 2007).

In this paper we report on the quality of outpatient paediatric malaria case-management in Kenyan health facilities, approximately 4–6 months after artemether–lumefantrine (AL) was introduced to replace sulfadoxine–pyrimethamine (SP) as the nationally recommended first-line therapy for uncomplicated malaria.

## Methods

### AL implementation activities in Kenya

The process surrounding the decision to abandon SP in favour of AL, and the main characteristics of the implementation process are presented in detail elsewhere (Amin *et al.* 2007). In summary, the key activities of the implementation process, important to effectively manage children with AL, comprised: (1) revision of national malaria case-management guidelines; (2) provision of three-day, theoretical, cascade, in-service training for health workers; (3) AL supply to the facilities; and (4) the distribution of job aids (guidelines and wall charts).

### Study design and data collection

Between 12 October and 19 December 2006, we studied outpatient malaria management using a cross-sectional, cluster sample survey at all government health facilities in four Kenyan districts (Kwale, Bondo, Kisii/Gucha and Makueni). A cluster was defined as all outpatient encounters between a health worker and patients on a single working day during the survey period. One survey day was randomly allocated to each facility and all patients presenting to outpatient departments of the surveyed facilities were recruited on the survey day. By the time of the surveys all districts had received at least initial supplies of AL and an in-service training programme for health workers had been completed. In this paper we describe case-management practices in children below 5 years of age, while data on patients 5 years and above will be presented elsewhere.

Data were collected by six teams at health facilities, each composed of two nurses, using three methods. First, all caretakers of sick children were interviewed when they exited the health facility. Prior to the interviews, they provided written informed consent to participate in the study. Interviewers collected information about the child's age, history of fever, main complaints, prior use of antimalarial drugs and if the visit was an initial or follow-up consultation. Information was collected from patient-held records about diagnostic procedures requested and results reported, medications prescribed, and if the patient was treated as an outpatient or referred for hospitalization. These records are the main communication link between clinicians, laboratories and pharmacies within Kenyan facilities. Furthermore, caretakers were also interviewed about counselling and drug dispensing practices undertaken during the facility visit. During the exit interview, all children had weight and axillary temperature measured by study nurses. Second, at the end of the day interviews were conducted with health workers who had previously attended sick children to collect information on their demographics, pre-service training, working experience, access to national guidelines, in-service training, exposure to supervision and knowledge about antimalarial treatment policy. If more than one health worker saw children at a facility, all were interviewed. Finally, a health facility assessment was performed to record the availability of supplies related to malaria case-management with an emphasis on stocks of antimalarial drugs, availability of malaria diagnostics and malaria treatment wall charts.

### Definitions

Our definitions reflected diagnosis, treatment, drug dispensing and counselling recommendations from the new *National Guideline for Diagnosis, Treatment and Prevention of Malaria for Health Workers in Kenya* (Ministry of Health (MoH) 2006a). According to this guideline and in line with Integrated Management of Childhood Illnesses (IMCI) recommendations (Ministry of Health (MoH) 2000), any child below 5 years of age with fever or history of fever in the absence of signs of severe malaria is presumed to have uncomplicated malaria in high malaria risk areas. For clinical management purposes all study districts are classified as a high risk area and the presence of history of fever despite the presence of other signs presents sufficient criterion to suspect and treat for malaria (Ministry of Health (MoH) 2000, 2006a). Although these recommendations do not consider parasitological diagnosis, the guideline in this patient group ambiguously states that *parasitological diagnosis is not pre-requisite for the treatment* (Ministry of Health (MoH) 2006a). To address this ambiguity we defined a case of uncomplicated malaria as a child who presented to a health facility for an initial visit with a history of fever during the present illness or axillary temperature ≥37.5 °C, and who was treated as an outpatient in the absence of a negative malaria test.

For uncomplicated malaria, we defined recommended treatment as prescription of AL for children ≥5 kg and quinine for children <5 kg. The correctness of AL dosage prescriptions was assessed based on weight-specific categories and classified into three categories: (1) recommended (six tablets for a child weighing 5–14 kg and 12 tablets for a child weighing 15–24 kg), (2) overdosed and (3) underdosed prescriptions. The quality of AL dispensing and counselling for patients included performance of the following tasks: weighing of patients, administration of the first dose at the health facility, observation of swallowing of the first dose, explanation on how to take AL at home, advising on taking AL after a meal and provision of advice on what to do in case of vomiting.

### Data entry and statistical analysis

Data were double entered by independent data entry clerks and verified using Access 2000 (Microsoft Inc., Redmond, WA, USA). The analysis was performed using stata, version 8 (StataCorp, College Station, TX, USA). The precision of proportions [95% confidence interval (CI)] was estimated accounting for the cluster sampling design using ‘health facility-day’ as the primary sampling unit.

We applied a stepwise approach in descriptive analysis of the quality of malaria case-management. Our primary analysis focused on the antimalarial treatment practices for children with uncomplicated malaria who needed to have AL prescribed. To assess overall performance of the new policy and to assess health workers’ adherence to the new treatment policy, analysis was undertaken at all health facilities and then limited to facilities with AL in stock on the survey day. Then, the quality of AL dosage prescriptions, drug dispensing and counselling received by children with uncomplicated malaria at facilities with available AL who had this drug prescribed was analysed. For comparison purposes this analysis was also undertaken on children meeting the same criteria but who had amodiaquine prescribed.

Finally, to explore factors influencing AL prescriptions, we analysed a series of health facility, health worker and patient level factors applying logistic regression modelling with the STATA *xtgee* procedure using an exchangeable working correlation matrix (Horton & Lipsitz 1999). This procedure uses generalized estimating equations to account for the correlated nature of the data. The analysis was restricted to children with an antimalarial drug prescribed at facilities with AL in stock. In the univariate analysis we estimated odds ratios (OR), *P*-values and 95% CIs for the association between health workers’ decision to prescribe AL and the following factors: health workers’ pre-service training; in-service training on the use of AL; access to national malaria guidelines; availability of amodiaquine; presence of AL wall charts; supervision including AL prescription; presence of positive malaria test; the child's temperature; and the child's main complaints. Factors with *P*-value <0.15 were entered into multivariate model. Hypothesis testing and CI estimation were done with an alpha level of 0.05.

### Ethical approval

Ethical approval for this study was provided by the KEMRI national ethical review committee (reference number 892) and by the Boston University Institutional Review Board (2004-127B and H-24055).

## Results

### Description of the sample

We evaluated outpatient malaria management at 193 health facilities where 228 health workers performed outpatient consultations on the day of the survey. Of 228 health workers, one (0.4%) left the facility before an interview could be performed and could not be found over the next 2 days. There were 1533 consultations evaluated for children <5 years of age and no caretaker of the sick children refused participation in the study. Of 1533 sick-child consultations, 983 met our definition of uncomplicated malaria. The other 550 consultations were follow-up visits, had neither a history of fever nor temperature ≥37.5 °C, or had negative blood slides reported at the health facility. A further seven children were excluded from the analysis because they did not have a weight measured. Of the remaining 976 children with uncomplicated malaria, 940 (96.3%) weighed ≥5 kg.

### Health facility and health worker readiness to deliver AL

Of 193 health facilities assessed, 172 (89%) had received AL with a median number of 48 days (interquartile range: 37–54) since the first receipt. On the day of the survey, any tablets of AL were available in 169 (88%) facilities; however, the availability of weight-specific AL blister packages ranged from 59% for 24 tablet packs to 87% for 12 tablet packs (Table 1). All 193 facilities stocked amodiaquine, nearly all had SP (95%) and quinine tablets were available in 80% of facilities. The facilities were well equipped with weighing scales (99%) and thermometers (80%); however, only 36% of facilities had the capacity to perform a parasitological diagnosis of malaria, more commonly using microscopy (29%) than rapid diagnostic tests (10%). Parasitological capacity to diagnose malaria was available in all hospitals, 77% of health centres and 20% of dispensaries. Only one facility had displayed AL case-management wall charts.

**Table 1:** Characteristics of the health facilities and health workers in four districts in Kenya

Characteristics	All districts, *n* (%)
**Health facility characteristics (*n* = 193)**	
Health facility type	
Hospital	14 (7.3)
Health centre	35 (18.1)
Dispensary	144 (74.6)
Equipment and services at health facility	
Weighing scale	191 (99.0)
Thermometer	155 (80.3)
Functional microscopy	55 (28.5)
Malaria rapid diagnostic test	19 (9.8)
Any malaria diagnostic service (rapid diagnostic test or microscopy)	70 (36.3)
Wall charts	
Artemether–lumefantrine case-management chart	1 (0.5)
IMCI chart	5 (2.6)
Availability of artemether–lumefantrine on the survey day	
Any tablets of artemether–lumefantrine	169 (87.6)
Artemether–lumefantrine six tablets pack	163 (84.5)
Artemether–lumefantrine 12 tablets pack	168 (87.1)
Artemether–lumefantrine 18 tablets pack	160 (82.9)
Artemether–lumefantrine 24 tablets pack	114 (59.1)
Availability of other antimalarial drugs onthe survey day	
Amodiaquine (any formulation)	193 (100)
Sulfadoxine–pyrimethamine (any formulation)	183 (94.8)
Quinine (tablets)	154 (79.8)
**Health worker characteristics (*n* = 227)**	
Pre-service training	
Doctor	2 (0.9)
Clinical officer	45 (19.8)
Nurse	150 (66.1)
Cadre without formal clinical training*	30 (13.2)
In-service training	
Malaria case-management training on artemether–lumefantrine	103 (45.4)
IMCI training	51 (22.5)
IMCI training including artemether–lumefantrine	6 (2.6)
Any training including artemether–lumefantrine (malaria-specific or IMCI)	105 (46.3)
Access to guidelines	
National malaria case-management guideline	126 (55.5)
IMCI guideline	65 (28.6)
Any supervisory visit including appropriateuse of artemether–lumefantrine	17 (7.5)
Knowledge of new treatment policy	
Treatment above 5 kg (artemether–lumefantrine)	158 (69.6)
Treatment below 5 kg (quinine)	69 (30.4)

IMCI, Integrated Management of Childhood Illnesses.

*This category includes 15 nursing aides, 11 community health workers, two public health technicians and two clerks.

Of 227 health workers interviewed, 105 (46%) had received in-service training on AL use, most commonly through the cascade malaria case-management training (103/105; 98%) (Table 2). Fifty-five per cent of health workers had access to a copy of the new national malaria case-management guideline recommending use of AL and 29% had IMCI guidelines that did not contain AL recommendations. Only 17 (8%) health workers reported a supervisory visit that included discussions on appropriate use of AL. Although only half of the health workers interviewed had received in-service training, 70% responded correctly that AL is the recommended first-line treatment for uncomplicated malaria in patients weighing >5 kg, whereas only 30% provided the correct response for children weighing <5 kg (quinine).

**Table 2:** Antimalarial treatments for children weighing ≥5 kg with uncomplicated malaria in Kenya

	All facilities (*n* = 940)		Facilities with AL in stock (*n* = 866)	
		
	*n* (%)	95% CI	*n* (%)	95% CI
AL	248 (26.4)	19.9–32.9	243 (28.1)	21.2–34.9
AQ	363 (38.6)	32.5–44.8	318 (36.7)	30.3–43.1
SP	41 (4.4)	1.5–7.2	28 (3.2)	1.3–5.2
AQ + SP	26 (2.8)	1.4–5.4	26 (3.0)	0.2–5.8
Quinine	14 (1.5)	0.5–2.4	13 (1.5)	0.5–2.5
Other antimalarial treatments*	31 (3.3)	1.7–4.9	29 (3.4)	1.6–5.1
No antimalarial prescribed	217 (23.1)	18.9–27.3	209 (24.1)	19.7–28.6

AL, artemether–lumefantrine; AQ, amodiaquine; SP, sulfadoxine–pyrimethamine; QN, quinine.

* Other antimalarial treatments include dehydroartemisinin, AQ + QN, SP + QN, AL + QN and AQ + SP + QN.

### Antimalarial treatments for children with uncomplicated malaria

Table 2 shows antimalarial treatments for children who needed AL prescribed at all facilities studied and separately at facilities where AL was in stock on the day of the survey. At all health facilities AL was prescribed for only 26% of children, amodiaquine for 39%, SP for 4%, various other antimalarials for 8% of children and, importantly, 23% of children left the facility without any antimalarial drug prescribed. Of 36 children <5 kg who were excluded from Table 2 and needed oral quinine according to national guidelines, 17 had amodiaquine prescribed, four various other antimalarials and 15 were not provided an antimalarial; none of these children had oral quinine prescribed. Restricting the analysis to facilities where AL was in stock on the day of the survey, no significant changes in adherence to AL recommendations were observed: 28% of children had AL prescribed, amodiaquine continued to be the health workers’ preference and the pattern of other antimalarial prescriptions was the same as was observed in the descriptions of all facilities (Table 2).

### Quality of AL dosage prescriptions, drug dispensing and counselling

When AL was prescribed, health workers prescribed it in correct weight-specific dosage for 92% of children, while overdosed and underdosed AL prescriptions were rare (2% and 6%, respectively; Table 3). Indeed, correct weight-specific prescriptions of AL were far more common than those of amodiaquine, which was correctly prescribed for only 15% of children (Table 3). Two hundred and forty-one children who received AL at the health facility were advised and counselled with varying degrees of adherence to guideline recommendations. Yet, these tasks were performed more commonly when AL was prescribed than for children who were prescribed amodiaquine (Table 4).

**Table 3:** Correctness of artemether–lumefantrine and amodiaquine dosage prescriptions

	Recommended, *n* (%)	Overdose, *n* (%)	Underdose, *n* (%)	Dosage not specified
Artemether–lumefantrine				
All children ≥5 kg (*n* = 248)	228 (91.9)	5 (2.0)	14 (5.7)	1 (0.4)
5–14 kg (*n* = 220)	212 (96.4)	5 (2.3)	2 (0.9)	1 (0.5)
15–24 kg (*n* = 28)	16 (57.1)	0	12 (42.9)	0
Amodiaquine				
All children ≥ 5 kg (*n* = 363)	53 (14.6)	132 (36.4)	174 (47.9)	4 (1.1)
5–6 kg (*n* = 46)	6 (13.0)	18 (39.1)	21 (45.7)	1 (2.2)
7–9 kg (*n* = 144)	24 (16.7)	54 (37.5)	66 (45.8)	0
10–14 kg (*n* = 135)	19 (14.1)	48 (35.6)	65 (48.2)	3 (2.2)
15–18 kg (*n* = 32)	4 (12.5)	11 (34.4)	17 (53.1)	0
19–37 kg (*n* = 6)	0	1 (16.7)	5 (83.3)	0

**Table 4:** Dispensing and counselling practices for children who had artemether–lumefantrine and amodiaquine dispensed

	Artemether–lumefantrine (*n* = 241)		Amodiaquine (*n* = 357)	
		
	*n* (%)	95% CI	*n* (%)	95% CI
Weight measured	167 (69.3)	58.8–79.8	133 (37.3)	26.6–47.9
First dose given at the facility	92 (38.2)	24.8–51.6	17 (4.8)	2.0–7.5
Swallowing of first dose observed	83 (34.4)	21.0–47.9	13 (3.6)	1.1–6.2
Dosage explained	234 (97.1)	95.0–99.2	346 (96.9)	95.0–98.8
Advice provided to take drug after a meal	91 (37.8)	28.0–47.5	n.a.	n.a.
Advice provided what to do in case of vomiting	19 (7.9)	3.8–11.9	19 (5.3)	2.0–8.7

n.a., not applicable.

### Predictors influencing AL prescriptions

We examined 13 factors that might have influenced prescribing of AL in 657 children with uncomplicated malaria who were prescribed an antimalarial at facilities where AL was in stock on the day of the survey. After univariate analysis the following factors did not meet entrance criteria (*P*-value >0.15) for the multivariate model: health worker's supervision including AL prescription (OR = 1.90; 95% CI: 0.65–5.61), main complaints of cough (OR = 0.93; 95% CI: 0.73–1.18), diarrhoea (OR = 1.20; 95% CI: 0.92–1.58) and ear problem (OR = 0.74; 95% CI: 0.32–1.70). In the multivariate model the following factors were significantly associated (*P*-value <0.05) with higher likelihood of AL prescription: health worker's pre-service training, in-service training including AL use, presence of a positive malaria test, main complaint of fever and temperature ≥37.5 °C (Table 5). In the same model, health worker's access to national guidelines and main complaints of skin problem were not significantly associated with measured outcome. The factors such as availability of amodiaquine and the presence of AL wall charts could not be analysed because they were either universally present or absent in the data set.

**Table 5:** Factors influencing health worker's decision to prescribe artemether–lumefantrine: results of multivariate analysis

Factor	No. of consultations	No. (%) with artemether–lumefantrine prescribed	OR (95% CI)	*P*-value
Health worker's pre-service training				
Clinical officer	112	71 (63.4)	5.10 (1.10–23.68)	0.038
Nurse	404	162 (40.1)	3.65 (0.97–13.66)	0.055
No formal training	141	10 (7.1)	1.0 (Ref.)	
Health worker received in-service training on artemether–lumefantrine				
Yes	266	155 (58.3)	2.58 (1.20–5.52)	0.014
No	391	88 (22.5)	1.0 (Ref.)
Health worker has access to national malaria guideline				
Yes	407	182 (44.7)	1.58 (0.74–3.37)	0.234
No	250	61 (24.4)	1.0 (Ref.)
Positive malaria test				
Yes	49	42 (85.7)	10.59 (2.62–42.73)	0.001
No	608	201 (33.1)	1.0 (Ref.)
Fever main complaint				
Yes	601	231 (38.4)	2.14 (1.17–3.91)	0.014
No	56	12 (21.4)	1.0 (Ref.)
Temperature ≥37.5 °C				
Yes	225	108 (48.0)	1.37 (1.03–1.82)	0.030
No	432	135 (31.3)	1.0 (Ref.)
Skin problem main complaint				
Yes	68	22 (32.4)	0.75 (0.49–1.16)	0.203
No	589	221 (37.5)	1.0 (Ref.)

## Discussion

Regular supply of adequate quantities of AL is crucial to any effective implementation plan. The results of our survey suggest that the majority (88%) of health facilities stocked AL; however, this remains far from perfect as one in ten facilities did not have any AL. The short interval between rolling out the AL drug supply and timing of our survey does not allow for any meaningful evaluation of the stability of the AL supply chain and estimates of stock-out durations. Nevertheless the drug supply challenges not only relate to the adequate supply of AL but also to the removal of ineffective drugs from the supply system. Our results show that amodiaquine was in stock in all facilities, a finding which is at variance with the stated national AL implementation plan recommending phasing-out of this drug (Ministry of Health (MoH) 2006b).

The Kenyan MoH set a target of 60% of front-line health workers to be trained before the delivery of AL to the health facilities (Amin *et al.* 2007). Across the four districts included in the present study, approximately 2 months after the delivery of AL to facilities, only 46% of health workers had received any specific training in the new guidelines. Notably 2 years after the delivery of AL to facilities in Zambia only 41% of health workers had received any training in the use of AL (Zurovac *et al.* 2007). The initial training exercise in Kenya cost approximately 1.5 million USD (Amin *et al.* 2007). To expand coverage of in-service training quickly and efficiently, cheaper, simplified and shortened on-job training options should be considered using, for example, trained clinical supervisors at the district level or communication of case-management messages through job aids.

By the time of our survey the distribution of job aids to support training and implementation of new policy had achieved mixed results. While 55% of health workers had access to a copy of the new guidelines either distributed during the training programmes or independently, only one health facility had newly produced AL case-management wall charts. Although the availability of wall charts showed mixed results on malaria treatment practices in the past (Zurovac & Rowe 2006), it is worrying that, by the time of the study, this intervention, which was already developed at the national level, did not reach health workers at the periphery of the health system. As they are a simple, inexpensive intervention to improve prescribing practices, the use of wall charts should be optimally implemented.

Despite some successes in delivery of AL to facilities and provision of in-service training, many children who should have received AL either were not treated or received suboptimal therapy with an alternative drug. Health workers had discontinued using SP (4%) but had an increased tendency to prescribe amodiaquine (39%) rather than AL (26%). While this practice is understandable at facilities without AL in stock, it is worrying that the same findings were revealed in facilities where AL was available. Furthermore, almost one in four children who should have received an antimalarial drug left the facility without any antimalarial. Similar findings (23%) were reported under AL treatment policy in Zambia (Zurovac *et al.* 2007). Compared to the results of the previous surveys undertaken under SP policy in Kenya (9%) (Zurovac *et al.* 2004) and chloroquine policy in Benin (6%) (Rowe *et al.* 2003), it appears that these practices are on the rise under the new AL policy. Further case-management interventions will need to pay particular attention to ensure that all febrile children receive an effective antimalarial treatment as recommended by national and international guidelines (Gove 1997; World Health Organisation (WHO) 2006).

More positively, when health workers decided to prescribe AL the quality of correct dosage prescription, drug dispensing and counselling was generally better for the new, more complex to use AL, than for more the familiar, less complex to use amodiaquine (Tables 3 and 4). These findings are encouraging in the light of previous findings under monotherapies that commonly reported incorrect dosing (Font *et al.* 2001; Nshakira *et al.* 2002; Zurovac *et al.* 2005b), poorer drug dispensing and counselling (Rowe *et al.* 2001; Armstrong Schellenberg *et al.* 2004; Zurovac *et al.* 2005b) and it was expected that the challenges under AL policy would be even greater (Zurovac & Rowe 2006). We are glad to report that our earlier concerns seem to be unfounded, most likely due to deployment of weight-specific blister packaging of AL providing clear dosing instructions not only to patients but also to health workers.

Our factor analysis provides some explanations that help understand deficiencies in prescribing antimalarial drugs. AL was more commonly prescribed by higher clinical cadres such as clinical officers than by nurses and health workers without formal clinical training. The opposite was observed during the era of SP policy in Kenya (Zurovac *et al.* 2004) and chloroquine policy in Benin (Rowe *et al.* 2003). One explanation is that there was some uncertainty among the Kenyan legislation revisions to allow prescribing of AL for lower cadres of health workers such as nurses (Amin *et al.* 2007). Furthermore, our findings reveal that not only nurses but also health workers without formal clinical training commonly perform consultations at the periphery of formal health system, although they are not officially allowed to do so. This problem was not anticipated during the AL implementation process and should be urgently addressed. Common programmatic interventions such as in-service training of health workers, distribution of guidelines and supervision including AL were variably associated with better prescribing practices in facilities where AL was in stock (Table 5). This has been an area of conflicting results in other settings; not all routine in-service initiatives to improve clinical management practices have had positive effects on treatment practices (Ross-Degnan *et al.* 1997; Rowe *et al.* 2000, 2001, 2003; World Health Organisation (WHO) 2001; Naimoli *et al.* 2006; Osterholt *et al.* 2006; Zurovac & Rowe 2006). In the present study perhaps the most important observation was that health workers who had been exposed to in-service training were three times more likely to prescribe AL to febrile children compared with those who had not received any training. As such this intervention worked but reached only 46% of health workers.

A series of patient factors deserve special attention. Children with a positive malaria test were 11 times more likely to have AL prescribed compared with children who were not tested. One possible explanation is that given the emphasis during training on the ‘rational use’ of AL through the use of diagnostics in adults, this message may have influenced the stated policy of presumptive treatment with AL for children. Our analysis further demonstrated that children presenting with a main complaint of fever or increased temperature were more likely to have AL prescribed, similar to reports from elsewhere in Africa (Rowe *et al.* 2003; Zurovac *et al.* 2004; Osterholt *et al.* 2006). This suggests that health workers might consider fever less significant in children, meriting less effective treatment, if temperature is not increased or if caretakers do not voluntarily report fever. Such practices may fail to result in the appropriate treatment of malaria. Health workers should be instructed to treat all fevers equally since it is well known that diagnostic reliance on only obvious fevers is likely to miss malaria diagnoses in young children (Gove 1997; World Health Organisation (WHO) 2006).

Finally, a series of subtle qualitative factors could not be examined in this study. For example, AL is an expensive drug on the private market and, although it is provided for free by the government, the subjective perceptions of health workers of AL as an expensive drug may influence prescribing. Furthermore, some of the case-management messages, such as those on the inefficacy of amodiaquine, use of malaria tests and presumptive diagnosis among others, may not have not have reached health workers at the periphery. In-depth investigation of these potential hypotheses using qualitative research methods is underway.

The Kenyan experience reported here, after only a few months of AL policy implementation, suggests that the changes in clinical practices at the point of care might take longer than anticipated. The constant, uninterrupted supply of drugs to facilities will ultimately determine how well the new policy is effective in reaching those in need of these medicines in the formal health sector. This obvious pre-requisite, however needs to be augmented by programme activities that withdraw ineffective medicines, train health workers in new guidelines, improve quality of supervision and constantly monitor ambiguities and buried messages in guidelines that effect correct prescription. Finally, it is also time to evaluate new, potentially cheaper and more effective approaches (e.g. provision of on-job training, delivery of non-monetary incentives, application of case-management reminders using text-messaging to health workers mobile phones) that might change clinical practice among health workers at the periphery of the health system.
